# Reproducibility of a web-based FFQ for 13- to 15-year-old Danish adolescents

**DOI:** 10.1017/jns.2015.39

**Published:** 2016-01-29

**Authors:** Anne A. Bjerregaard, Inge Tetens, Sjurdur F. Olsen, Thorhallur I. Halldorsson

**Affiliations:** 1 Center for Fetal Programming, Department of Epidemiology Research, Statens Serum Institut, Copenhagen, Denmark; 2 Risk–Benefit Research Group, National Food Institute, Technical University of Denmark, Søborg, Denmark; 3 The Unit for Nutrition Research, Faculty of Food Science and Nutrition, School of Health Sciences, University of Iceland, Reykjavik, Iceland

**Keywords:** FFQ, Reproducibility, Web-based FFQ, Dietary assessment, Adolescents, GDM, gestational diabetes mellitus, ICC, intra-class correlation coefficient

## Abstract

FFQ are widely used in large-scale studies to assess dietary intake. To aid interpretation of diet–disease associations assessment of validity must be performed. Reproducibility is one aspect of validity focusing on the stability of repeated assessment with the same method which may also reveal problems in instrument design or participant instructions. The aim of the present study was to evaluate the reproducibility of a web-based FFQ targeting Danish adolescents within the Danish National Birth Cohort (DNBC). Data for the present study were obtained from a prospective design nested within the DNBC. Adolescents aged 13 to 15 years old (*n* 48, 60 % girls) completed the FFQ twice 4 weeks apart. The proportion of adolescents consistently classified into the same tertile according to amount of food intake ranged from 45 % (fish) to 77 % (vegetables), whereas classification into opposite tertiles ranged from 0 % (fruit, oils and dressing) to 15 % (beverages). Overall, no significant differences were observed in intake of food groups or nutrients between the two completions of the FFQ. Mean crude Spearman correlation for all food groups was 0·56 and mean intra-class correlation for all food groups was 0·61. In conclusion, the reproducibility of the FFQ for Danish adolescents was acceptable. The study revealed that adolescents aged 13–15 years seemed capable of recalling consistently overall dietary habits and had some difficulties estimating the frequency of consumption of regularly consumed food items.

FFQ are widely used in large-scale studies to assess dietary intake. The FFQ is relatively inexpensive when self-administered, less intrusive and less time-consuming for the participants compared with other dietary assessment methods such as 24-h recalls and food records^(^^1^^–^^3^^)^. Due to the design of an FFQ with a list of food items and response categories of how frequently the food items are consumed, the FFQ is appropriate for ranking individuals into groups of habitual dietary intake. As a result, the FFQ is primarily used to study associations between habitual dietary intake and diseases in epidemiological studies^(^^4^^–^^6^^)^.

To detect possible associations between diet and diseases it is crucial to clarify if the dietary assessment method is valid. One aspect of validity is to evaluate the reproducibility of the method^(^^7^^–^^9^^)^. Reproducibility gives an indication of whether the dietary assessment method will generate similar results independently of researcher and occasion when distributed several times and may reveal problems in instrument design or respondent instructions^(^^10^^)^. When evaluating reproducibility the interval between occasions is an important factor to consider as too long intervals may simply reflect true change in dietary intake in addition to normal variation in responses, whereas a short temporal separation may reflect carry-over responses from previous completion of the FFQ^(^^11^^)^.

Even among adults, completing an FFQ can be a difficult cognitive task, as it requires accuracy in terms of memory, ability to identify various types of foods and their preparation methods as well as quantification of the amounts consumed^(^^12^^,^^13^^)^. In children, it is generally assumed that from the age of around 10 years they can recognise and describe quantities necessary for filling out an FFQ^(^^13^^)^. However, adolescents are more prone to omit and intrude foods when reporting their diet compared with adults^(^^13^^–^^15^^)^. Even though skills in estimating portion sizes increase with age, focus on body image which seems to affect the accuracy of adolescents’ self-reported dietary intake also increases with age^(^^16^^,^^17^^)^. For adolescents, previous studies report that foods consumed on a regular basis and main courses are more accurately reported compared with foods eaten less often^(^^14^^,^^18^^,^^19^^)^. However, lack of attention among adolescents may increase recall bias^(^^17^^,^^20^^)^. To increase motivation among adolescents and reduce costs of distribution and processing data, web-based FFQ have been developed by several research groups^(^^21^^–^^23^^)^. An acceptable level of reproducibility and validity of such FFQ have been observed among adolescents^(^^21^^,^^22^^,^^24^^)^.

In spite of the limitations and potential pitfalls mentioned, adolescents are generally considered able to recall dietary intake at an acceptable level as they have knowledge of food items, the concept of time, and portion size estimation^(^^13^^)^. It is, however, for this age group extremely important to elucidate the validity and reproducibility of the FFQ designed for adolescents to facilitate further development of instruments that can be used to accurately assess the diets of adolescents. The aim of the present study was to evaluate the reproducibility of a web-based FFQ targeting Danish adolescents, aged 13–15 years, within the Danish National Birth Cohort (DNBC).

## Methods

### Subjects and study design

The DNBC was initiated in 1996 and includes information from 101 042 pregnancies. Various data from gestation week 12 and 30, and 6 and 18 months postpartum are available^(^^25^^)^. Detailed offspring follow-up was conducted at the ages of 7 and 11 years.

Data for the present study were obtained from a subcohort study within the DNBC, which ran from May 2012 to April 2014. The purpose of this study was to investigate insulin sensitivity, risk of type 2 diabetes after pregnancy, and risk of diabetes in a group of children aged 9–15 years whose mothers had had gestational diabetes mellitus (GDM) and in a similar age group of offspring of women who had had a normal pregnancy^(^^26^^)^. Enrolled adolescents aged 13–15 years of age who completed a web-based FFQ (FFQ1) at a clinic visit were invited for this reproducibility study. No exclusion criteria were included because the invited adolescents were relatively healthy in terms of non-communicable diseases. Invitations were distributed to 100 adolescents born to both GDM and non-GDM mothers and were handed out at the clinic between January and March 2013. The adolescents were asked to complete a second FFQ (FFQ2) at home 4 weeks after the clinic visit. At the clinic they could ask for help from parent or clinical personnel if needed. The FFQ was available online and the adolescents entered the FFQ with a personalised ID number and password. Follow-up on completing FFQ2 was done first by email and second by mobile text message. Time for filling in the FFQ was approximately 40 min.

The GDM study was approved by the National Committee on Health Research Ethics in Denmark (H-4-2011-045).

### FFQ

The web-based FFQ was developed from the validated Youth/Adolescent Questionnaire (YAQ) used in the US Growing Up Today Study (GUTS)^(^^27^^,^^28^^)^. The YAQ was translated into Danish and modified to include typical Danish foods based on reports on National Danish Dietary Habits and Physical Activity^(^^29^^,^^30^^)^. The FFQ is based on the online html program Limesurvey. Screen dumps from the FFQ are available (www.ssi.dk^(^^31^^)^).

The FFQ included frequency questions about 145 food items and the recall period was the previous month. The food items were clustered into the following eight groups (number of food items in parentheses): beverages (18); dairy products (8); bread and cereals including butter on bread (13); spread on bread (14); cold and warm dishes (25); side dishes and condiments (18); fruit and vegetables (29); snacks and desserts (20). Frequency scales used for beverages, milk products, bread, side dishes and condiments, added salt and sugar, fruit and vegetables ranged from ‘did not drink/consume the last month’ to ‘4 times or more per d’. For cereals, spread on bread, cold and warm dishes, snacks and desserts, the frequency scale ranged from ‘did not consume the last month’ to ‘2 times or more per d’. Predefined portion sizes based on standard portions developed by the National Food Institute in Denmark^(^^32^^)^ were applied for beverages and fruit, whereas for the rest of the food items no portion size was given. For calculations of intake in g per d all frequencies were computed into times per d and multiplied with standard portion sizes. Foodcalc v.1.3 combined with the Danish Food Table was used for estimating food and nutrient intake^(^^33^^,^^34^^)^.

Open-ended questions were included to record additional information on food allergy, foods the participant may avoid, and questions about changes in food habits in the last month. Furthermore, the FFQ contained questions on (number of questions in parentheses): age, sex, self-reported height and weight, meal habits (15), physical activity (11) and puberty (6). Small notes and pop-up questions (16) were given throughout the questionnaire in order for the participant to keep the recall period in mind. Written instructions on how to complete the questionnaire were available on the first two pages of the questionnaire together with examples.

## Statistics

Descriptive analyses were performed to evaluate participant charateristics. Age- and sex-specific BMI was determined based on the International Obesity Task Force^(^^35^^)^. Overweight was defined as BMI > 25 kg/m^2^ and obesity as BMI > 30 kg/m^2(^^35^^)^. The highest level of education of two parents was divided into four levels: more than 4 years of post-secondary education as high level of education (corresponding to a masters or PhD level), 3–4 years of post-secondary education as medium level of education (corresponding to bachelors level), skilled workers (9 years of basic school plus 5 years of vocational training), and unskilled workers.

Deviation from normality for dietary variables was evaluated by QQ-plots and histograms. Because the majority of variables were skewed, all variables are presented as medians with 25th and 75th percentiles. Differences between groups were examined using the non-parametric Wilcoxon sum-rank test. Correlation coefficients estimated between the two FFQ were Spearman's correlation coefficients and intra-class correlation coefficients (ICC). Cohen's weighted κ statistics and misclassification analyses (into thirds) were applied to test the FFQ's ability to rank individuals according to intake of food groups, energy and macronutrients.

To examine how consistently subjects could answer simple questions covering school lunch, supplement use, leisure time physical activity and selected food items, κ statistics were applied. The selection of food items for this analysis was based on the most popular and unpopular food items among 11- to 14-year-olds described in the report Dietary Patterns of Danish Children and Adolescents 2000–2004^(^^29^^)^. The popularity of food items was determined by summarising how frequently food items were consumed over a period of 7 d and thereby comparing high-frequency food items with low-frequency food items^(^^29^^)^. The purpose of this analysis was to examine if the adolescents are able to consistently answer simple questions on their overall dietary habits/lifestyle and still have sufficient knowledge/awareness of the foods even though they have difficulties in estimating frequency of consumption. Thus, with irregular eating habits among adolescents, 100 % agreement between measures of specific food items could not be expected. Sensitivity analyses were performed for children born to mothers with and without GDM, age-specific BMI and supervision from parents.

All statistical analyses were performed in the statistical program SAS version 9.4 (SAS Institute).

## Results

### Subjects

A total of fifty-two subjects agreed to participate. Four individuals did not complete the second questionnaire although several reminders were provided which resulted in forty-eight subjects completing the study. One subject was excluded from further analyses due to too many questions being omitted in the questionnaire which resulted in extremely low energy intake in FFQ2 (energy intake < 2500 kJ/d). Table 1 shows participant characteristics with mean age being 13·5 (sd 0·58) years and median BMI 19·7 (5th–95th percentiles 17–28) kg/m^2^. Age- and sex-specific BMI by the International Obesity Task Force categorised eleven subjects as overweight and three subjects as obese^(^^33^^)^. The majority of participants were girls (60 %) and 60 % were born to mothers with GDM (61 % girls). Comparing those who completed both FFQ (*n* 48) with non-participants (*n* 52), respectively, no substantial difference was found for proportions of girls, age, BMI, born to GDM mothers, or parental educational level (data not shown).

**Table 1.** Participant characteristics (*n* 47, 60 % girls)

	*n*	%
Age (years)		
Mean	13·5	
sd	0·6	
BMI (kg/m^2^)		
Median	19·7	
5th–95th percentiles	17–28	
Overweight (*n* (%))*	11	23·4
Obese†	3	6·4
Children born from GDM mothers	28	59·5
Parental educational level‡		
High level of education		21·0
Medium level of education		36·0
Skilled workers		21·0
Unskilled workers		21·0

GDM, gestational diabetes mellitus.

* Overweight: BMI >25 kg/m^2^.

† Obese: BMI >30 kg/m^2^.

‡ Highest educational level of two parents during pregnancy presented as percentage.

### Dietary intake

Table 2 presents the median intake of food groups, energy and macronutrients in FFQ1 and FFQ2. No significant differences were observed between food groups or nutrients in FFQ1 compared with FFQ2. The percentage difference between FFQ1 and FFQ2 showed that the overall intake reported tended to be higher in FFQ1 compared with FFQ2; however, only beverages, meat/poultry, fish and fat intake reached a difference of more than 20 %. The tendency of higher intakes reported in FFQ1 compared with FFQ2 for food groups was confirmed using Bland–Altman plots where the mean difference between FFQ1 and FFQ2 was above zero in all food groups (data not shown). Crude Spearman correlations and ICC for food groups ranged from 0·39 (beverages) to 0·83 (fruit) and from 0·37 (beverages) to 0·83 (fruit), respectively. Lower ICC compared with Spearman correlations for most food groups except for bread, fish, fruit and carbohydrate showed some within-subject variance. Adjustment by sex and BMI resulted in stronger Spearman correlations for a few food groups and most profoundly for cereals, vegetables and protein (g/d) (data not shown). The proportion of subjects classified correctly into the same tertile for food groups ranged from 45 % (fish) to 77 % (vegetables), whereas the proportion of subjects misclassified (into the opposite tertile) ranged from 0 % (fruit, oils and dressing) to 15 % (beverages). Furthermore, Cohen's weighted κ values ranged from 0·23 (fish) to 0·71 (vegetables) (see Table 2).

**Table 2.** Differences in and intra-class correlations for food groups and energy and macronutrients between FFQ1 and FFQ2 (*n* 47)

	FFQ1		FFQ2						Classified into tertiles (%)		
	Median*	p25–p75th	Median*	p25–p75	*P*†	FFQ1/FFQ2 (%)	Spearman correlation: *r*‡	Intra-class correlation: *r*§	Same	Opposite	Cohen's weighted κ
Food groups											
Beverages (g/d)	1019	632–1205	759	538–1166	0·11	34	0·39	0·37	47	15	0·30
Dairy products (g/d)	350	208–710	343	154–561	0·18	2	0·56	0·43	68	6	0·57
Bread (g/d)	189	129–286	181	130–252	0·63	4	0·56	0·71	53	9	0·37
Cereals (g/d)	31	14–53	32	14–52	0·93	−3	0·55	0·45	66	4	0·57
Meat/poultry (g/d)	85	56–136	70	54–150	0·16	21	0·54	0·51	53	9	0·37
Fish (g/d)	45	7–17	10	5–15	0·34	350	0·42	0·61	45	13	0·23
Fruit (g/d)	167	42–260	144	50–286	0·69	16	0·83	0·83	62	0	0·57
Vegetables (g/d)	124	72–188	105	57–170	0·41	18	0·71	0·68	77	2	0·71
Sugar and sweets (g/d)	54	37–105	51	33–114	0·78	6	0·71	0·62	62	4	0·52
Oils and dressing (g/d)	33	18–48	31	18–43	0·77	6	0·72	0·66	66	0	0·61
Nutrients											
Energy (MJ/d)	7·8	5·7–10·3	7·5	5·4–9·9	0·49	4	0·78	0·75	49	4	0·37
Protein (g/d)	73	54–99	69	50–88	0·18	6	0·69	0·64	49	4	0·37
Fat (g/d)	73	50–95	61	48–97	0·36	20	0·68	0·66	55	2	0·47
Carbohydrate (g/d)	217	177–346	257	162–312	0·69	−16	0·65	0·79	53	4	0·42
Protein (% energy)	15	2·4	15	2·1	0·05	0	0·35	0·33	43	11	0·23
Fat (% energy)	33	5·9	32	6·3	0·89	3	0·65	0·65	53	4	0·42
Carbohydrate (% energy)	52	6·1	53	6·7	0·36	−2	0·52	0·51	55	6	0·42

p25, 25th Percentile; p75, 75th percentile.

* Median and percentiles for all variables except energy percentage from macronutirents: mean and sd.

† Median difference for all variables except energy percentage from macronutirents: mean difference.

‡ *P* < 0·001 for all food groups.

§ Reliability test *P* < 0·05 for beverages, dairy products, cereals, energy percentage from protein, *P* < 0·0001 for all others.

We found no significant difference in food groups or energy and macronutrient intake between children born to mothers with/without GDM. An overall lower reported intake in all food groups was seen in adolescents with age- and sex-specific BMI > 25 kg/m^2^ (*n* 14) compared with adolescents with age- and sex-specific BMI < 25 kg/m^2^ (*n* 33); however, the difference was only significant for sugar and sweets (mean difference 43·6 (95 % CI 4·08, 83·1) g/d; *P* = 0·03). FFQ1 was self-reported by 38 % of the participants whereas FFQ2 was self-reported by 81 % (*P* < 0·0001). The proportion of girls was similar to the proportion of boys who received support from parents in FFQ1 (girls 61 % *v*. boys 58 %). The same was seen for the proportion receiving parental support in FFQ2 (girls 18 % *v*. boys 21 %).We found no significant difference in energy intake between levels of support.

Agreement with respect to specific questions about dietary habits and selected food items between FFQ1 and FFQ2 is presented in Table 3. The first questions listed in the table focus on dietary habits whereas the next questions concern frequently consumed, rarely consumed and less healthy food items, respectively. The percentage agreement for dietary patterns (89–94 %) showed that the adolescents to a high degree were able to recall overall dietary behaviour. For regularly consumed food items such as white bread (34 %), whole-grain bread (36 %) and potatoes (34 %), agreement was lower compared with more rarely consumed foods such as fish (62 %), porridge (68 %) and avocado (70 %). However, at the same time a relatively accurate recall was seen for type of milk (83 %) and type of dairy products (79 %). Lower agreement was also seen for less healthy food items such as Nutella (40 %) and jam (32 %).

**Table 3.** Agreement in questions between FFQ1 and FFQ2 (*n* 47)

Selected questions	Complete agreement between FFQ1 and FFQ2 (%)
Dietary patterns/lifestyle	
From where did you get lunch most often on school days the last month?	89
Did you consume multivitamins?	94
Did you do any sport exercise in your spare time?	98
Type of milk	83
Type of dairy products	79
Frequently consumed food items	
White bread	34
Whole-grain bread	36
Cornflakes	53
Potatoes	34
Rarely consumed food items	
Fish	62
Porridge	68
Sausages	55
Avocado	70
Less healthy food items	
Sugar-sweetened beverages	55
Nutella	40
Jam	32
Candy	58

## Discussion

Using an FFQ designed to capture dietary habits of Danish adolescents we found that the FFQ categorised adolescents consistently (47–77 %) according to the magnitude of food intake at two different occasions, making the method reproducible at an acceptable level^(^^36^^)^. For beverages, fish and energy percentage from protein, correlation coefficients, classification into tertiles, and Cohen's weighted κ were lower compared with the other variables. This highlights the need for cautious interpretation when considering the validity of future association studies based on these variables. Furthermore, we elucidated that adolescents were able to accurately report overall dietary habits, but had some difficulties reporting regularly consumed food items, which demand a more specific estimation of frequency.

Our findings are comparable with studies including the adolescent population^(^^23^^,^^37^^)^. Xia *et al*.^(^^37^^)^ included 168 adolescent girls from North China aged 12–18 years with 9 months between administrations of an eighty-one-item FFQ. They reported higher percentage for correct classification into quartiles for food groups (64·5–83·9 %) compared with our study. However, in a study of fifty-eight adolescents from Norway aged 13–14 years who completed a 131-item FFQ 4 weeks apart, Overby *et al*.^(^^23^^)^ found between 36 % (bread and cereals) and 55 % (dairy products) correct classification of subjects into quartiles. The lower level of correct classification compared with our study was not expected because the applied FFQ was developed based on the Danish FFQ and the methodology was somewhat similar between the two studies. Nevertheless, in our study correct classification was above 50 % and Cohen's weighted κ values were above 0·4 which has been suggested to be acceptable for reproducibility studies in adults^(^^36^^,^^38^^)^. Additionally, the number of food items in the FFQ, sample size and number of categories in the classification analysis could affect the level of correct classification. Spearman correlation coefficients and ICC for both food groups and nutrients were comparable with or higher than those found in other studies among adults and adolescents^(^^37^^,^^39^^,^^40^^)^. Overby *et al*.^(^^23^^)^ showed correlation coefficients between 0·41 and 0·67 for food groups and 0·57 to 0·62 for macronutrients, whereas Xia *et al*.^(^^37^^)^ reported ICC between 0·58 and 0·73 for food groups. However, in a review Cade *et al*.^(^^8^^)^ reported that the most common correlations in reproducibility studies are between 0·5 and 0·7 among adults. Overall, the reproducibility found in the present study was acceptable and comparable with other studies including adolescents even though differences in population size, methodology and design of FFQ differ somewhat between studies.

The overall decrease in reported intake of specific food groups from FFQ1 to FFQ2 in our study is similar to what has been reported by others^(^^22^^,^^23^^,^^37^^,^^41^^)^ and could partly be due to the fact that FFQ2 to a greater extent was completed without parental support. Learning effects from FFQ1 to FFQ2 could also reduce reported intake in FFQ2^(^^1^^)^. It could also be speculated that completing the second FFQ at home alone would decrease focus and increase omission of some food items. In both the studies by Overby *et al*.^(^^23^^)^ and Rockett *et al*.^(^^2^,^7^^)^, the FFQ was self-administered at both occasions. However, even in the studies by Xia *et al*.^(^^37^^)^ and Filippi *et al*.^(^^22^^)^ where the FFQ was administered in class with adult assistance, the intake of most food groups decreased from FFQ1 to FFQ2.

The comparison between specific questions to examine consistency between FFQ1 and FFQ2 showed good agreement for overall dietary habits and certain food products. Similar results were reported in a US study including 415 children aged 11–12 years completing an FFQ with twenty-five food items focusing on foods contributing to fat, fibre, fruit and vegetable intakes^(^^42^^)^. Their item-to-item comparison revealed higher agreement for single listed and regularly eaten foods such as milk compared with items listed with multiple foods (e.g. doughnuts, sweets rolls or muffins)^(^^42^^)^. Similar results were reported in another study from the USA including 259 children from the 8th grade who completed food and meal behaviour questions 7–14 d apart. The results showed 79–96 % agreement for school lunch, supplement use, type of milk and physical activity^(^^43^^)^. The lower level of agreement for specific and regularly consumed food items observed in our study supports the possibility that adolescents have some difficulties registering frequency. Higher correlation coefficients for food items consumed with low frequency indicate that these items are easier to recall. Frank *et al*.^(^^44^^)^ support this finding as they reported higher correlation coefficients for rarely consumed food items among 1108 American adolescents. On the one hand, this could be because it is easier to answer ‘no’ to consumption rather than estimating a specific frequency. It could also be due to multiple food items listed in one question (e.g. ‘Cornflakes, Bran-flakes, Special-K etc.’) compared with single listed food (e.g. ‘avocado’). The lower level of agreement for less healthy food items could be explained by these food items being ‘not socially acceptable’ among adolescents. However, it could also be that these food items may actually be consumed on a regular basis^(^^29^^)^.

There are a number of potential strengths and limitations in the present study. First, all parental educational groups were well represented, which is rarely seen within these types of studies. In large cohort studies, higher socio-economic status is often over-represented. Second, the design with a web-based FFQ was easily accessible for the participants and the 4-week time interval seemed adequate as the observed degree of reproducibility may reflect variation in responses rather than changes in dietary intake^(^^11^^)^. The size of the study population is a potential limitation to recognise together with that fact that 60 % of the study population were children born to GDM mothers. This resulted in a relatively high proportion of overweight children, which may make the results less representative for the Danish adolescent population^(^^45^^,^^46^^)^.

### Conclusion

In conclusion, the web-based FFQ for Danish adolescents was reproducible to an acceptable level with observed classification analyses and correlation coefficients comparable with similar studies in adolescents. In addition, the study revealed that the adolescents seemed capable of consistently recalling overall dietary habits and had some difficulties estimating the frequency of consumption of regularly consumed food items. The reported level of reproducibility is important when assessing dietary intake among adolescents to facilitate the development of instruments for accurate assessment of the diet of adolescents.
